# Evaluation of OrthoChromic OC-1 films for photon radiotherapy application

**DOI:** 10.1093/jrr/rrac080

**Published:** 2022-12-01

**Authors:** Seng Boh Lim, Grace Tang

**Affiliations:** Department of Medical Physics, Memorial Sloan Kettering Cancer Center, 1275 York Ave, New York, New York 10065, USA; Department of Medical Physics, Memorial Sloan Kettering Cancer Center, 1275 York Ave, New York, New York 10065, USA

**Keywords:** film dosimetry, quality assurance, radiotherapy, orthochromic film

## Abstract

A new film dosimetry system consists of the new OrthoChromic™ OC-1 film, and a novel calibration procedure was evaluated. Two films, C1 and C2, were exposed simultaneously using the 6FFF beam with a step-wedge pattern of five steps ranging from 590 to 3000 cGy. C1 was used for calibration, and C2 was used for calibration curve validation. The second scan of C2 was done by rotating the film by 90-deg. To evaluate the effectiveness of the non-uniform scanner response correction with the new system, a film was exposed to a 20 × 20 cm^2^ field. The beam profile measured with the film was compared to the IBA cc04 measurements in water. Films were irradiated to characterize the energy response, dynamic range and temporal growth effect. Open (MLC-defined) and clinical fields were radiated to evaluate the overall performance of the new system. The new calibration procedure was validated with an average dose difference of 1.6% and a gamma (2%,2 mm) passing rate of 100%. With C2 scanned 90-deg rotated, the average dose difference was 1.3%. The average difference between cc04 and film was 0.4%. The S_t_ between films and diode/cc04 were within −0.3% difference for 1 × 1 to 14 × 14 cm^2^ and −2.8% for 0.5 × 0.5 cm^2^. For clinical fields, the average gamma (3%,2 mm) was 98.8%. These results were consistent with EBT3 film and MapCheck measurements with a dose > 400 cGy. The results have shown that the OC-1 film system can achieve accurate results for QA measurements, but more considerable uncertainty was observed within the low dose range.

## INTRODUCTION

A film dosimetry system consists of two components, film and scanner, and both parts can contribute to errors. Radiochromic film is an excellent QA tool but is prone to unexpected dosimetric uncertainty if not handled with care. An example of a common and significant dosimetric error is when films are scanned 90 deg rotated from the calibration films, resulting in a systematic shift in dose [1,2] of the order of 10%. Another source of uncertainty arises from the non-uniform lateral response of the scanner [3–5]. The triple-channel calibration was proposed to minimize some artifacts by optimizing the calibration curves generated by the red, green and blue optical channels [6]. The single-scan calibration protocol [7], which incorporates triple-channel calibration, was proposed to streamline the workflow and minimize the scanner-induced dosimetric variation. Note that while all three-color channels were utilized to generate the calibration curve, calibrated films were analyzed using only one-color channel, and the color channel used depends on the dynamic range of the films. Considering these challenges, a typical film dosimetry workflow can be labor-intensive and requires care to attain reasonable accuracy.

The new OrthoChromic™ (OrthoChromic, Hillsborough, NJ, USA) film dosimetry system attempts to resolve these significant sources of error with the new OrthoChromic OC-1 film and a novel calibration procedure [8]. Unlike the conventional radiochromic films constructed with an active layer sandwiched between substrates, the active layer of OC-1 film is deposited on top of a single layer of polyester substrate [9]. With reflection scanning, an attempt to reduce the light travel to polarizing polymer interfaces, the dosimetric discrepancies from films scanned with different orientations can be significantly reduced. On the other hand, the variation in lateral response of the scanner is corrected concurrently with the intensity-to-dose conversion during the calibration step using the OrthoChromic Pro software. While the applications of this film for ultra-violet and proton radiation were recently reported with promising results [9,10], to our best knowledge, this is the first report on the fundamental performance of this new film dosimetry system for photon radiation therapy dosimetry.

## METHOD

**Fig. 1:**
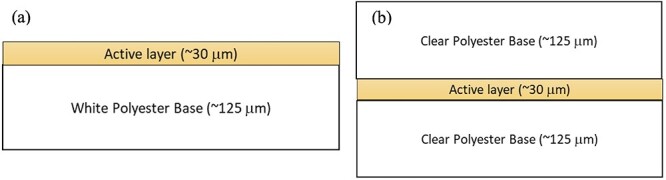
The schematic of the film structure of (a) Orthochromic film and (b) Gafchromic EBT 3 film.

OrthoChromic OC-1 film is constructed with the active layer coated on a white polyester base (Fig. 1a), requiring reflective scanning. The active layer is sandwiched between two clear polyester bases for the conventional Gafchromic EBT3 type film (Fig. 1b), requiring transmission scanning.

Film measurements were performed with Solid Water slab phantoms (Sun Nuclear, Melbourne, FL, USA) on a Varian TrueBeam linac (Varian Medical Systems, Palo Alto, CA, USA). All films were scanned with 150 dpi in reflection mode using an Epson 12000XL scanner and analyzed using in-house software. Except in the temporal growth study, films were scanned 24 hours after exposure to minimize the potential growth variation. The uncertainties were measured with the one standard deviation (k = 1) of the measurements in line with AAPM TG-235 [2]. The batch numbers for the OC-1 and EBT3 were 2-200 310-1 and 07301901, respectively.

### Validation of the novel calibration procedure

The calibration procedure involved intensity-to-dose conversion and scanner spatial correction using the OrthoChrome-Pro software. Different from some other film dosimetry software, OrthoChrome-Pro only accepts DICOM dose files as input for the generation of calibration curves. To convert intensity to dose, a treatment plan was created in the treatment planning system (TPS) that emulates the delivery of the calibration film [8]. Two films, C1 and C2, were exposed simultaneously using the 6FFF (flattening-filter free) beam with a step-wedge pattern of five 15 × 3 cm^2^ steps ranging from 590 to 3000 cGy at 5 cm depth and SAD 100 cm. 6FFF beam is chosen because of its non-flat dose profile, providing more data points to the film calibration curve (dose vs gray scale) with a single field compared to flattened beams.

The delivery was achieved by moving the couch with the pre-defined shifts in the TPS. The software first registered the scanned calibration film with the TPS calculation. The calibration curve was generated by matching each calculation voxel with the corresponding scanned calibration film. The fields were defined by the multileaf collimators (MLC) and the couch was advanced to different positions for each step field such that each step field has the same beam profile but with different doses. C1 was used for calibration and C2 was used to verify the calibration curves for systematic errors. To evaluate the effectiveness of the correction for non-uniform scanner response, a film was exposed with an open 20 × 20 cm^2^ field at SSD 100 cm and 5 cm depth. With and without applying the lateral response correction in the calibration software, the calibrated film profiles were extracted from the same film and compared to that measured with an IBA cc04 ion chamber (IBA Dosimetry GmbH, Schwarzenbruck, Germany) in water. Gamma analysis [11], γ, with the [12] dosimetric and distance to agreement criteria of 2% and 2 mm was generated using the OrthoChrome software. Here, the threshold was set to 5.0%.

### Characteristics of OC1-film

#### Scan orientation dependence

To assess the scan orientation dependence of the OC-1 film, the same film used for calibration verification, C2, was scanned again but with 90 deg rotation. The dose was then compared with the dose from the original scan, where the film was not rotated. In both cases, the lateral correction was activated in the software.

#### Energy response

The energy dependence of the OC-1 film was evaluated for three photon beams and five electron beams: 6 MV, 6FFF MV, 15 MV, 6 MeV, 9 MeV, 12 MeV, 16 MeV and 20 MeV.

Two pieces of OC-1 film were exposed per beam energy at a depth of maximum dose, d_max_, with a 10 × 10 cm^2^ field and 1000 MU (see Table 1).

**Table 1:** Summary of the dmax of all beam energies used for energy dependence assessment

Beam energy	6X	6FFF	15X	6E	9E	12E	16E	20E
d_max_ (cm)	1.5	1.5	3.0	1.5	2.0	2.5	2.5	2.5

The step-wedge calibration film was exposed using the 6FFF beam on the same day. The output of each energy was measured with a calibrated Exradin® A12 ion chamber (Standard Imaging, Middleton, WI, USA) in the same set of Solid Water phantom. The outputs of each energy, *E_i_*, as measured by A12 and OC-1 were defined as, ${D}_{A12}({E}_i)$, and ${D}_{OC-1}({E}_i)$, respectively. The relative outputs, ${D}_{A12}^{6 FFF}({E}_i)$, and ${D}_{OC-1}^{6 FFF}({E}_i)$ as measured by A12 and OC-1 respectively, were defined as the ratio between the ${D}_{A12}({E}_i)$, and ${D}_{OC-1}({E}_i)$, and the corresponding 6FFF outputs ${D}_{A12}(6 FFF)$ and ${D}_{OC-1}(6 FFF)$. The relative output ratio, R_output_(E_i_), of each energy, between the OC-1 and ion chamber was calculated as follows:

(1) \begin{equation*} {R}_{output}\left({E}_i\right)=\frac{D_{OC-1}^{6 FFF}\left({E}_i\right)}{D_{A12}^{6 FFF}\left({E}_i\right)} \end{equation*}

The energy response of OC-1 was assessed by the degree of deviation of this ratio from unity.

#### Dynamic range

The dynamic range of the OC-1 film was explored by exposing the films with 0 cGy to 12 000 cGy using 6 MV with an open 10 × 10 cm^2^ field at 1.5 cm depth and 100 cm SAD. Each dose level was exposed to two pieces of OC-1 film. The net optical density (OD) [2] of each channel was calculated and plotted with the corresponding dose level [13]. A fitting function relating OD and dose, D, was used to generate the relationship [14]. The OrthoChromic film exhibited a similar behavior to a logarithmic curve rather than a power curve [14]. The data were fitted using a logarithmic curve fitting function as shown below.

(2) \begin{equation*} OD=a\cdot{\ln}\left(D+\gamma \right)+b{D}^m \end{equation*}

where a, b, γ and m are fitting parameters. The sensitivity [14], S, was then determined by taking the derivative of [2] with respect to D:

(3) \begin{equation*} S=\frac{a}{D+\gamma }+ mb{D}^{m-1} \end{equation*}

#### Temporal growth effect

The temporal growth effect of the OC-1 film was evaluated with two pieces of film that were exposed with 1000 cGy using the 6 MV beam with an open 10 × 10 cm^2^ field at 1.5 cm depth and 100 cm SAD. The exposed films were scanned at 0.5, 1, 2, 4, 8, 16, 32, 64, 128 and 250 hours after irradiation. Per scan instance t, each of the two films was scanned twice. The average raw reading of the two films, R(t), for each scan instance was generated by taking the average values of a 1 × 1 cm^2^ ROI at the center of the field of each of the two films. The temporal net OD(t) [2] at each scan instance t was obtained with the following expression:

(4) \begin{equation*} OD(t)={\log}\left(\frac{2^{16}}{R(t)}\right) \end{equation*}

The temporal relationship of OD(t) was then characterized with the power curve fitting:

(5) \begin{equation*} OD(t)={c}_1{e}^{-\tau t}+{c}_2{t}^n \end{equation*}

where t is time in hour, c_1_, c_2_, τ and n are fitting constants. The rate of OD(t) change can be determined by:

(6) \begin{equation*} \frac{dOD(t)}{dt}=-{c}_1\tau{e}^{-\tau t}+{c}_2n{t}^{n-1} \end{equation*}

### Open and clinical fields

#### Open fields

To assess the film response with different open fields, the output factors, S_t_, were generated by exposing films with 0.5 × 0.5 to 14 × 14 cm^2^ at SSD 100 cm and 10 cm depth. The film S_t_ were compared with the IBA stereotactic diode (SD) and cc04 daisy-chained measurements in water. The measured S_t_ were corrected with the detector specific factors [1].

#### Clinical cases

Three clinical cases (1 VMAT and 2 IMRT) were measured with OC-1 films, EBT3 films and SRS MapCHECK/MapCHECK2 (Sun Nuclear, Melbourne, FL). Details of the plan can be found in Table 2.

**Table 2:** Plan information of clinical cases

Case	Site	Prescription	Beam energy
VMAT 1	Multi-Met SRS	23 Gy × 1	6FFF
IMRT 2	Lung	8 Gy × 5	6 MV
IMRT 3	Lung	4 Gy × 5	6 MV

One to two fields of each plan were measured. The calibration film of each case extended about 10–20% higher than the maximum dose of the case to anticipate the full dynamic dose range [2]. For EBT3, calibration was performed following our departmental procedure [15]. The calibration curve was generated with twelve points with a dose range from 0 cGy to 110% of the maximum dose of the clinical fields. Each calibration point was delivered with a 4 × 4 cm^2^ square field. The known doses of the calibration points were previously measured with an ion chamber in Solid Water phantom under the same condition. To minimize the scanner artifacts [3], the calibration film is cut into three strips along the scanning direction. Each strip is placed in the center and scanned. With this curve and triple channel calibration [6], the clinical films were calibrated.

All clinical films were exposed from two to three times to capture the dosimetry of the clinical fields within the film dynamic range. All measurements of the clinical fields were done with films placed in a 30 × 30 × 10 cm Solid Water phantom, at 5 cm depth with 5 cm backup, SAD 100 cm. The phantom was placed on the couch top. For the VMAT plan, the films were irradiated with gantry rotation and for IMRT plans, the films were exposed in the phantom with beams reset to gantry 0 deg. The VMAT plan was also measured with the SRS MapCHECK (with gantry rotation) and the IMRT plans were measured with MapCHECK2 (with gantry reset to 0 deg). All films and MapCHECK measurements were compared with the TPS dose distribution using local gamma analysis [11], γ, with the recommended [12] dosimetric and distance to agreement criteria of 3% and 2 mm based on a ROI defined by the 50% isodose line using our in-house film analysis software. The average dose within the ROI was first calculated. The dosimetric agreement metric was determined by taking 3% of the average dose. The TPS in this study was commissioned meeting the recommendations of the AAPM MPPG 5a [16], and the treatment unit was credentialed with IROC OSLD remote program [17]. Here, the TPS dose distribution was calculated with Eclipse v15.5 (Varian Medical Systems, Palo Alto, CA, USA) with the Anisotropic Analytical algorithm (AAA) [18] using a dose grid of 1.25 mm.

## RESULTS

### Validation of the novel calibration procedure

Compared to TPS, the average dose difference of C1, within the irradiated area of the film, was 0.1%, and the gamma passing rate of 100% based on a 2%/2 mm criteria, while the average dose difference between C1 and C2 was 1.6% with a gamma passing rate of 100%. The dose difference is similar to the radiochromic EBT3 dosimetric uncertainty of ~1.5% for photon beams [2]. Because C1 and C2 were exposed and scanned simultaneously (i.e. C1 and C2 were scanned with one single scan), the dose difference between these films was mainly due to film-to-film variation.

**Fig. 2:**
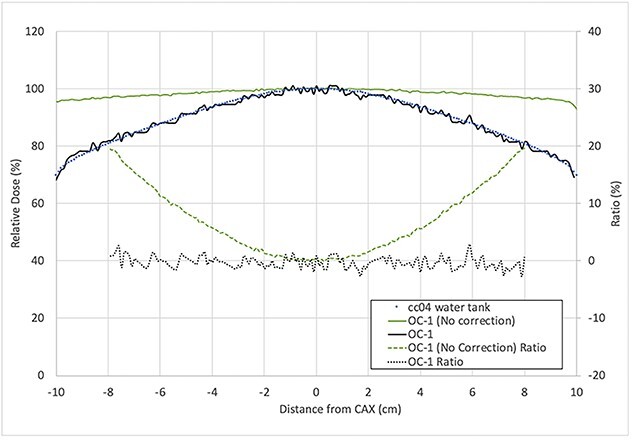
Profile comparison of cc04 measurement in water tank (blue circle), OC-1 film without correction (green line), and OC-1 film with full correction (black line) measurement for 6FFF beam, of a 20 × 20 cm^2^ field size at 5 cm depth and 100 cm SSD. The percentage ratio of the measurements is presented with the dashed lines.

The integrated scanner lateral response correction in the calibration procedure was found to be effective. The comparison of profiles measured by the IBA cc04 ion chamber in water and OC-1 film is shown in Fig. 2.

Note that each profile was normalized to its central axis dose. Without applying the lateral correction to the OC-1 measurement, a dose difference of more than 3.0% was observed starting at about +/− 2.8 cm from the central axis and continued to increase further away from the central axis, with an over-response of +19.6% at 8.0 cm. The average relative dose difference between film and cc04 within ~80% of the field width, which is typically used to define the beam flatness [19], was found to be 0.4% and 7.1% for the film with and without correction, respectively, indicating that the lateral scanner response correction from the novel calibration procedure was effective and without systematic error.

### Scan orientation dependence

When the C2 film was scanned without any rotation from the calibration film, the average dose discrepancy from TPS was 1.0% (range: −0.4% to 3.7%) for the five different dose levels at the central axis of each of the five step-field as shown in Table 3.

**Table 3:** Dosimetric effect of scan orientation based on the C2 film. C2(0°) was scanned without any rotation relative to the scan orientation of the calibration film and C2(90°) was scanned with 90 degrees rotation. Dose at the central axis (CAX) was compared

Step wedge	Dose at CAX (cGy)	Dose difference (%)		
		Film – TPS		
		C2 (0°)	C2 (90°)	C2 (90°) – C2 (0°)
1	3087	−0.4%	1.6%	1.2%
2	2523	1.7%	3.6%	1.9%
3	1924	0.2%	2.5%	2.3%
4	1312	−0.4%	2.2%	2.6%
5	700	3.7%	2.3%	−1.4%

When the same film was scanned 90 deg rotated from the calibration film scan orientation, the average dose error increased to 2.4% compared to TPS (range: 1.6% to 3.6%) and the average relative difference to the scan done without any rotation was 1.3% (range: −1.4% to 2.6%).

### Energy response

**Fig. 3:**
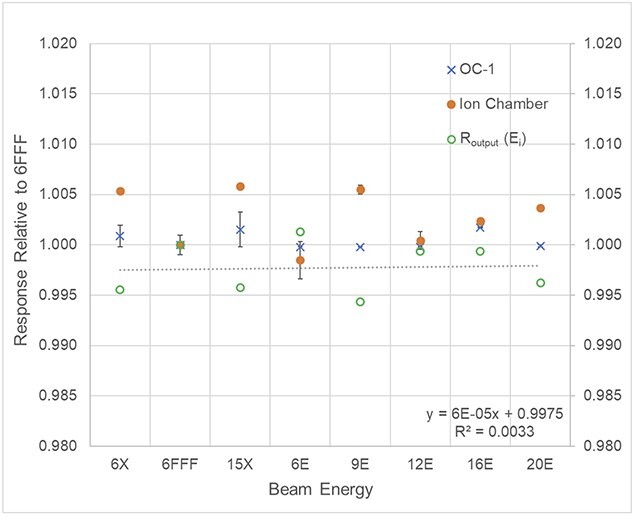
Energy dependence of OC-1 film compared to A12 ion chamber.

Fig. 3 shows the responses of OC-1 with different photon and electron beam energies. ${D}_{A12}^{6 FFF}({E}_i)$, measured with A12 ion chamber, were found to be between 0.998 to 1.005 cGy/MU, while ${D}_{OC-1}^{6 FFF}({E}_i)$, measured by OC-1, were in the range of 1.000 to 1.002 cGy/MU. Subsequently, R_output_(E_i_) were between 0.994 to 1.001 and no significant trend with beam energy was observed from the best-fit curve (R^2^ = 0.0033).

### Dynamic range

**Fig. 4:**
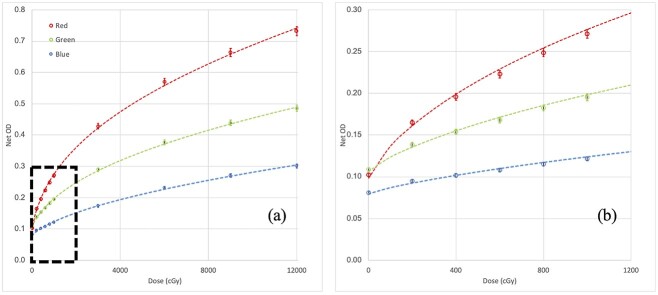
Net OD variation of the three color channels (red, green, blue) with dose (a) from 0.0 cGy to 12 000 cGy; (b) from 0.0 cGy to 1200 cGy*.*

The OD of OC-1 changed in the range of 0.102 to 0.731, 0.109 to 0.486 and 0.081 to 0.300 for red, green and blue channels, respectively, with irradiated doses from 0 to 12 000 cGy (Fig. 4).

**Fig. 5:**
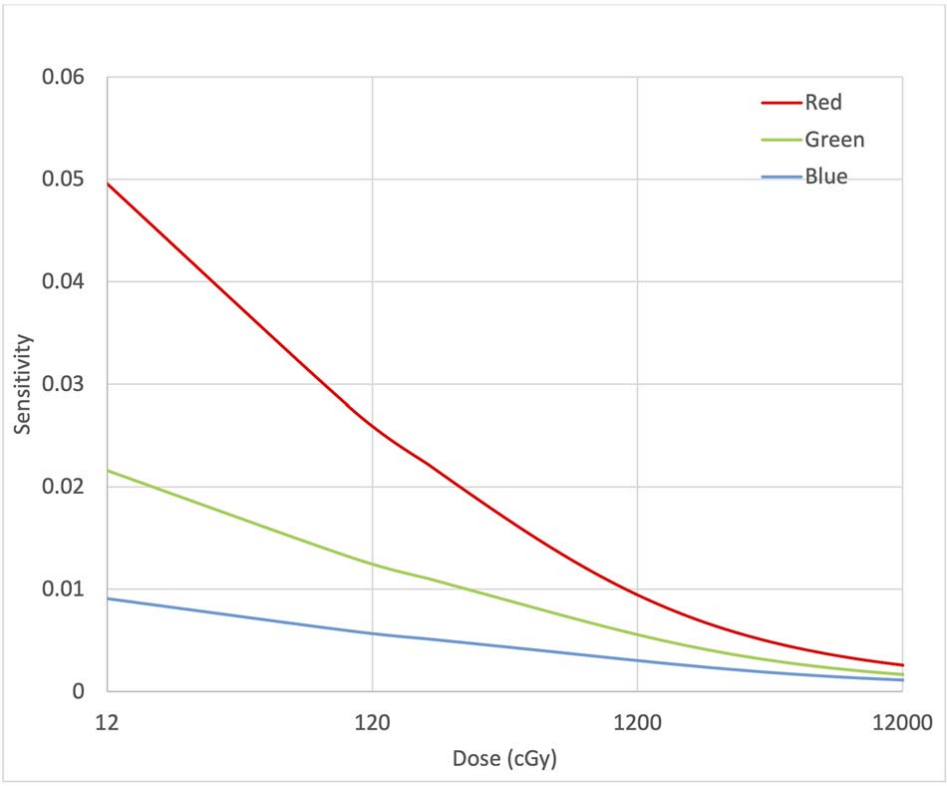
Sensitivity variation of the three channels for dose from 12 to 12 000 cGy.

The slopes of the OD curves flattened with increasing dose. The sensitivity of the red, green and blue channels changed from 51.5 × 10^−3^, 22.3 × 10^−3^ and 9.4 × 10^−3^ to 2.6 × 10^−3^, 1.7 × 10^-3^ and 1.1 × 10^−3^, respectively. Fig. 5 shows the sensitivity change of the three-color channels with dose. Among the three channels, the red channel exhibited the largest change in sensitivity from 0 to 1000 cGy followed by the green and blue channels.

### Temporal growth effect

**Fig. 6:**
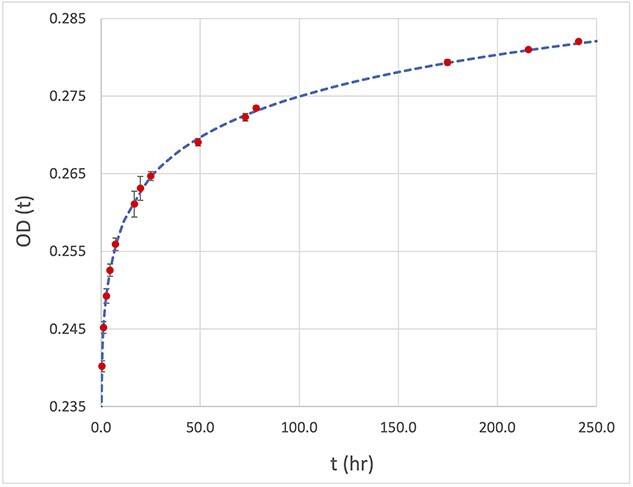
Temporal change of OD of OC-1 after irradiation.

The OD(t) changed from 0.240 at 0.40 hour to 0.282 at 240 hours after irradiation as shown in Fig. 6.

**Fig. 7:**
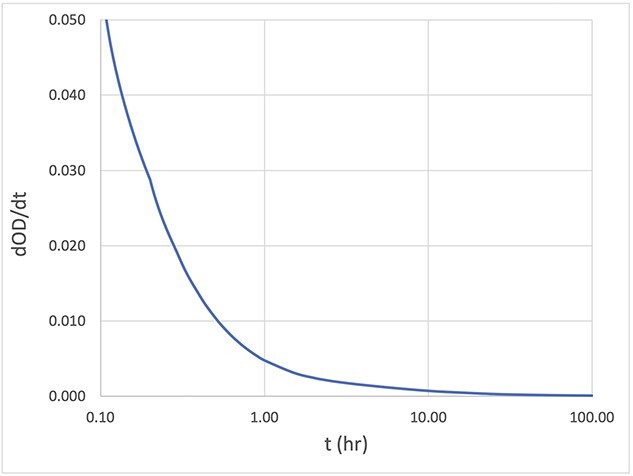
The rate of change of dOD/dt with respect to time.

The OD growth rate decreased from the initial rate of 1.35% per hour at 0.4 hours to 0.003% per hour at 240 hours after irradiation. Fig. 7 shows the rate of change in OD with time. At about 7 hours after irradiation, the rate of OD change dropped to 0.1% per hour.

### Open field measurements

**Fig. 8:**
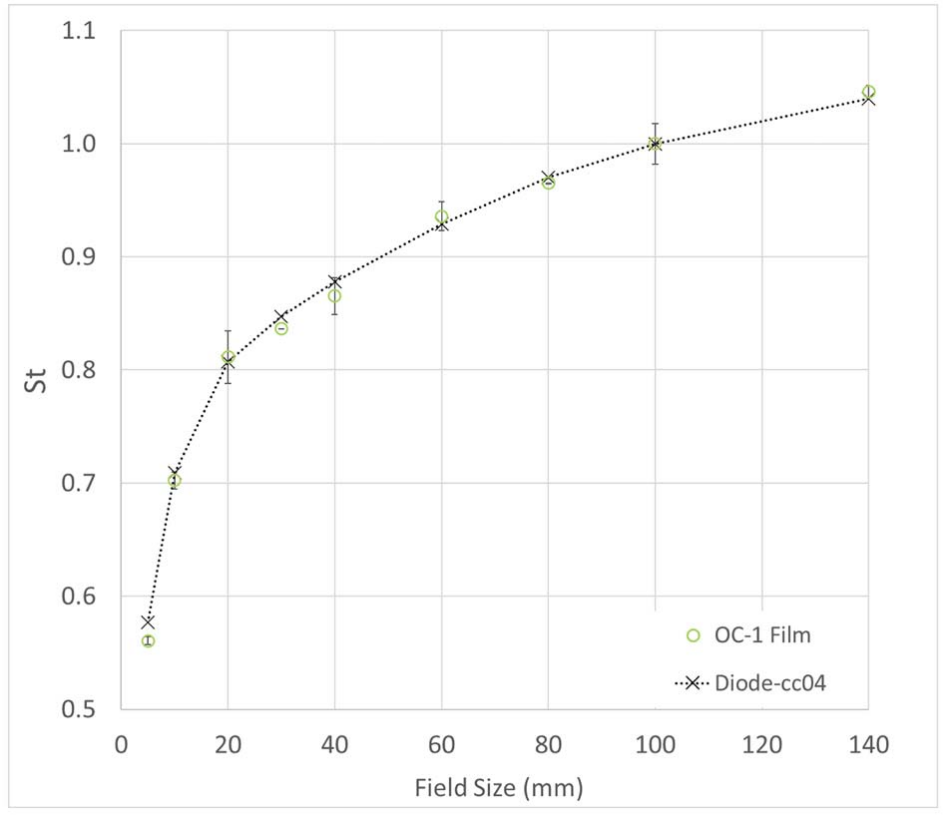
Comparison of total scatter factor, S_t_, measured by diode/cc04 in water tank (black cross/line) and OC-1 film (green circle) for the 6FFF beam at SSD 100 cm and depth of 10 cm.

Compared to the S_t_ measured with SD/cc04 in water, the average difference was within −0.3% (range: 0.5% to −1.4%) from 1 × 1 to 14 × 14 cm^2^ and −2.8% for 0.5 × 0.5 cm^2^ as shown in Fig. 8.

### Clinical case measurements

Table 4 shows the summary of the clinical case measurements. Compared to TPS, the average γ passing rate for OC-1 films was 98.6% (range: 95.8%-100.0%), 99.7% for EBT3 films (range: 99.3–100%) and 98.6% for SRS MapCHECK/MapCHECK2 (range: 95–100%). The results of the two-measurement set of IMRT cases were similar, with gamma passing rates of 98.9% and 100% and 98.8% and 99.3%. The OC-1 film measurements were consistent with the EBT3 film and SRS MapCHECK and MapCHECK2 measurements with fields with predominantly high dose. However, the OC-1 measurements were noticeably inferior to other detectors when there were significant portions of low dose.

## DISCUSSION

This work presented the evaluation results of the new OC-1 film dosimetry system with megavoltage photon and electron beams. The novel calibration procedure is fast and straightforward, and the correction of non-uniform scanner lateral response is a particularly useful feature. It is worth noting that this correction consists of two components – it corrects for the inherent film non-uniformity and the scanner lateral response, similar to the method developed by Lewis *et al*. [3]. However, the robustness of dose calibration relies on the quality of beam models in TPS. Currently, the software assumes perfect agreement between machine and TPS calculation. According to AAPM MPPG 5a [16], the tolerance of calculation accuracy is 2% for PDD and off-axis output factors compared to linac commissioning data. This means there can be a systematic error of up to 2% in dose calibration when the TPS was modeled following national standards. Currently, the software mandates using DICOM dose files for calibration, i.e. dose calculation from TPS. Therefore, we recommend that users double-check the accuracy of beam models using a large open field, such as comparing beam profiles with measurement, to better understand the intrinsic dose error in film calibration.

The new film construction significantly reduces the dosimetric error due to differences in scan orientation [2,13,20–22] by approximately 2.2%. This improvement is likely attributed to the combination of reflective scanning and eliminating a transmission clear polyester base of the OC-1 film. However, care should still be taken, and all films should be labeled correctly to reduce systematic error in the film dosimetry process. Aside from the construction, the OC-1 film shares some similarities with the EBT-type films. Regarding energy response, no energy dependency with the OC-1 film was found within measurement uncertainty, similar to radiochromic type [2,22] films. As for dynamic range, the net OD varies similarly with the high dose radiochromic EBT-XD film [2,22], which has a usable range [2] from 4 to 4000 cGy. The sensitivity decreased steadily from 800 cGy to 12 000 cGy, and the relative rates among the three channels were found to be constant. The sensitivity of the red channel changed rapidly at doses below 700 cGy. At doses below 100 cGy, the red channel attained a significantly higher sensitivity than the green and blue channels (Fig. 5). Even with triple channel optimization, which strives to reduce the film artifacts and disturbances [6], higher dosimetric uncertainty in sub-100 cGy has been in the order of 5-7% for radiochromic film as reported by Howard *et al* [23]. In this study, although the OC-1 films were scanned in the reflective mode to improve low dose performance [2], the higher red channel sensitivity relative to the green and blue channels in the low dose range (< 100 cGy) may post a challenge to the dose disturbance optimization and can be a contributing factor to the increased dose uncertainty. One remedy to such a challenge is to expose the same clinical field multiple times, effectively shifting the dose range of interest away from the higher uncertainty range. Further investigation is underway to understand better the dosimetric uncertainty seen at the low dose range.

Post-irradiation, OC-1 film exhibited growth at a rate greater than 5.0% per hr in OD, similar to the radiochromic film [2,7,13,22]. Twelve hours after irradiation, the OD growth was about 10.2%, but the growth rate decreased to 0.6% per hr. Although OD continued to grow even at 250 hr after irradiation, the rate of change was very slow at 0.03% per hr and 0.02% per hr at 24 hr and 48 hr post-irradiation, respectively. Coupled with the single-scan technique [2,7], the impact of OD growth can be easily managed if the scan is performed a few hours post-irradiation. In this study, the OD results did not investigate the OD growth of individual channels, which should be explored in future study.

**Table 4:** Measurements of clinical cases using OC-1 films, EBT3 films and SRS MapCHECK/MapCHECK2, compared to TPS based on a gamma criteria of 3%/2 mm. ROI is defined by the 50% isodose line

Case	Field	Dose in ROI(cGy)		# of film exposure	γ(3%, 2 mm)		
		Max	Average		OC-1	EBT3	SRS MapCHECK (VMAT) MapCHECK2 (IMRT)
VMAT 1	1	984.0	665.3	2	95.8%	99.7%	99.3%
IMRT 2	1	300.4	198.4	2	98.9%	99.3%	100%
				3	100%	-	
IMRT 3	1	261.0	201.4	2	98.8%	100%	95%
	2	300.0	220.0	3	99.3%	99.7%	100%

OC-1 films can be a good tool for stereotactic radiosurgery type measurements, based on the S_t_ comparison to the standard small field detectors such as SD. Even for a small field size of 0.5 × 0.5 cm^2^, the difference between OC-1 and SD was at a relatively reasonable value of 2.8%. For clinical cases, OC-1 films generally obtained similar results to EBT-3 film, especially for those fields within the higher dose range, but OC-1 film, similar to the high dose radiochromic film [24], exhibited more variation, particularly at dose < 100 cGy. This is likely related to the high OD change in the dose range of the clinical fields. This issue could be improved by increasing the exposure dose. In addition, the high uncertainty in lower dose ranges can be masked with stereotactic cases with prescription > 10 Gy using gamma analysis as doses < 10% are often not considered. Overall, the accuracy of OC-1 was comparable with the EBT-type film with a dose > 400 cGy.

In this work, only triple-channel calibration was explored. Single-channel calibration in the low dose range will be performed to assess the potential dose accuracy in future studies. This study was only conducted with a single batch of film and limited clinical cases. A more extensive study with multiple film batches and a more comprehensive range of clinical cases will also be done to assess the inter-batch variation and the patient-specific QA effectiveness.

## CONCLUSION

The new dosimetry system with OC-1 film was found to meet and possibly exceed the expectations of film dosimetry with a dose > 400 cGy. Ongoing work includes a further direct comparison with the conventional radiochromic film dosimetry system and the long-term reliability of the new film.
